# Soil Microbiome of Tropical Seasonal and Permanent Small Wetlands

**DOI:** 10.1111/1758-2229.70306

**Published:** 2026-03-30

**Authors:** Karen Luko‐Sulato, Everton Tiago Sulato, Jorge R. Osman, Pedro Nolasco‐Jiménez, Daniela Morales, Graziela Silva Rezende, Cassy Anne Rodrigues, Sandra Imaculada Maintinguer, Anderson Ferreira da Cunha, Vania Rosolen

**Affiliations:** ^1^ Departamento de Geologia, Instituto de Geociências e Ciências Exatas (IGCE), Universidade Estadual Paulista (UNESP) Rio Claro São Paulo Brazil; ^2^ Departamento de Engenharia Ambiental Instituto de Geociências e Ciências Exatas (IGCE), Universidade Estadual Paulista (UNESP) Rio Claro São Paulo Brazil; ^3^ Instituto de Geología Económica Aplicada (GEA), Universidad de Concepción Concepción Chile; ^4^ Instituto de Recursos Naturales y Agrobiología de Sevilla (IRNAS‐CSIC) Sevilla España; ^5^ Departamento de Genética e Evolução Universidade Federal de São Carlos São Carlos São Paulo Brazil; ^6^ Lab de Ecologia Vegetal, Universidade Estadual Paulista (UNESP), Instituto de Biociências Rio Claro Brazil; ^7^ Instituto de Pesquisa em Bioenergia (IPBEN) Rio Claro São Paulo Brazil

**Keywords:** *Archaea*, *Bacteria*, metabarcoding, soil edaphic factors, wetland

## Abstract

Characterisation of the microbial communities of two small tropical wetlands under two distinct hydrological regimes (permanent and seasonal), across a rainy and dry season was performed by 16S rRNA amplicon sequencing. We identified 48 bacterial phyla across the two wetland types, seasons and depths and 83% of the bacterial sequences consistently corresponded to six phyla: *Acidobacteria*, *Actinobacteria*, *Bacteroidetes*, *Chloroflexi*, *Proteobacteria* and *Verrucomicrobia*. The seasonal wetland presented a predominance of *Chloroflexi*, *Nitrospirae*, *Actinobacteria* and *Acidobacteria*, whereas the permanent wetland showed higher relative abundances of Planctomycetes, Bacteroidetes, Proteobacteria and Firmicutes. Archaeal communities also differed, with *Crenarchaeota* predominating in the seasonal and *Euryarchaeota* in the permanent wetland. Microbial communities showed pronounced compositional shifts across wetland type, season and depth, while maintaining stable alpha diversity, with depth was the dominant driver. Functional inference suggested that aerobic ammonia oxidation, nitrate reduction and sulphur compound respiration were the predominant putative metabolic pathways in the seasonal wetland and methanogenesis, fermentation, dark hydrogen oxidation, nitrogen fixation, photoautotrophy, ureolysis and hydrocarbon degradation in the permanent wetland. The permanent wetland exhibited sparse correlation with environmental drivers, consistent with long‐term saturation and chronic nutrient limitation, while the seasonal wetland presented influence of pH, nutrients and SOC on microbial community structure.

## Graphical abstract

Distinct hydrological regimes shape microbial communities and metabolic pathways in tropical wetlands. Redox‐driven shifts highlight enhanced methanogenesis under anoxia, and dominant archaeal and bacterial groups reflect specific redox conditions and adaptations, with important implications for biogeochemical cycling and climate‐relevant processes.

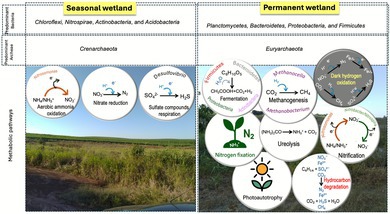

## 1 Introduction

Natural wetlands are complex terrestrial ecosystems, characterised by perennial or seasonal waterlogging, which cover 2.1 million km^2^, near 6% of the terrestrial surface (Zhang et al. 2016). These ecosystems encompass great diversity and importance, which provide many environmental services, as being nurseries for animal species (Uzarski et al. 2009) and acting as recharge points for surface waters (Furlan et al. 2020).

The soil microbiome facilitates the nutrient supply to plants and controls the fate of contaminants. However, human‐induced warming of climate may cause unprecedented changes in the physiological and metabolic activities of soil microbiomes. Changes in the diversity of soil microbiomes in turn alter the elemental pools and cycling in wetlands and have a direct and lasting impact on soil function and ecosystem service (Cavicchioli et al. 2019). As soil microbiomes encompass microorganisms with differences in physiology, growth rate and sensitivity to temperature (Dubey et al. 2019), any change in the composition of this microbiome will affect the cycling of elements sensitive to redox conditions and nutrients (Dong et al. 2024). Therefore, a robust understanding of the composition of the microbial consortium is needed to understand how the metabolic and physiological responses of microorganisms in different conditions explain the overall wetland health and enable predicting and managing ecosystem responses to environmental changes (Ligi et al. 2014; Peralta et al. 2013).

Among genomic approaches, metagenomics has emerged as a powerful tool for assessing microbial community structure and function by analysing the genetic material of all microorganisms present in a given environment (Hu et al. 2024). Several studies have applied this approach to wetlands, consistently highlighting how microbial communities are structured by hydrological regimes, edaphic factors and anthropogenic influences. Ligi et al. (2014), using high‐throughput 16S rRNA amplicon sequencing, characterised bacterial communities in the Olentangy River wetland complex, Ohio, reinforcing the critical role of hydrological regimes, as *Acidobacteria* and *Actinobacteria* were notably more abundant in wetlands with seasonal water regimes, particularly in transitional areas. Depth‐related variation in microbial composition has also been documented. Microbial community richness across peat bog profiles in Maine, USA, was generally higher at depths around 15 cm, with methanogens detected even in superficial layers (Arnold et al. 2023). Focusing on microbial metabolic functions, Dong et al. (2024) investigated microaerophilic Fe(II)‐oxidising bacteria in wetlands of Guangdong, China. Their findings underscored a strong correlation between Fe(III)‐reducing bacteria and carbon‐fixing microorganisms under anoxic conditions, suggesting intricate links between iron cycling and carbon sequestration processes.

Anthropogenic impacts are also key drivers of microbial community dynamics in wetlands. Zhang et al. (2024) documented a 40‐year microbial diversity restoration trajectory in the Poyang Lake wetland, China, following cessation of agricultural activities, where *Acidobacteria* abundance was fostered by agricultural practises, while *Gammaproteobacteria* became more prominent post‐agriculture. Peralta et al. (2013) investigated microbial communities in wetlands of the Piedmont region, Virginia and found that five bacterial phyla—*Acidobacteria, Actinobacteria, Bacteroidetes, Firmicutes and Proteobacteria*—comprised 80% of the microbial composition. Notably, in comparison to constructed ones, natural wetlands were not only more abundant in *Acidobacteria*; it was observed that this group tended to be more abundant as constructed wetlands became older. In an urban wetland in South Africa, *Proteobacteria, Bacteroidetes and Firmicutes* dominated microbiomes at sites heavily influenced by human activities (Koloti et al. 2024).

On this basis, the critical role of microbial communities in ecosystem functioning, as well as their high sensitivity to environmental changes, is well documented. In addition, small tropical wetlands, which serve as biodiversity hotspots and play important roles in global biogeochemical cycles, are particularly affected by hydrological regimes because their limited spatial extent increases their susceptibility to environmental fluctuations (Rosolen et al. 2019). Comparing permanent and seasonal wetlands is instrumental to assess how variations in water availability shape microbial composition and their interactions with environmental factors (Ligi et al. 2014). This understanding is crucial for predicting ecosystem responses to environmental changes, managing wetland biodiversity and preserving their ecological functions. We hypothesised that these distinct hydrological regimes and seasonal variation significantly shape microbial composition and diversity, primarily through shifts in redox conditions and nutrient availability in the superficial soil layers. Therefore, the objective of this study was to assess the interplay between environmental factors and microbial composition in two small tropical wetlands with contrasting hydrological regimes, one permanent and one seasonal. We aimed to explore how variations in water regimes, seasonal changes, two soil superficial depths and edaphic factors influence the microbial communities present.

## 2 Materials and Methods

### 2.1 Study Site

**FIGURE 1:**
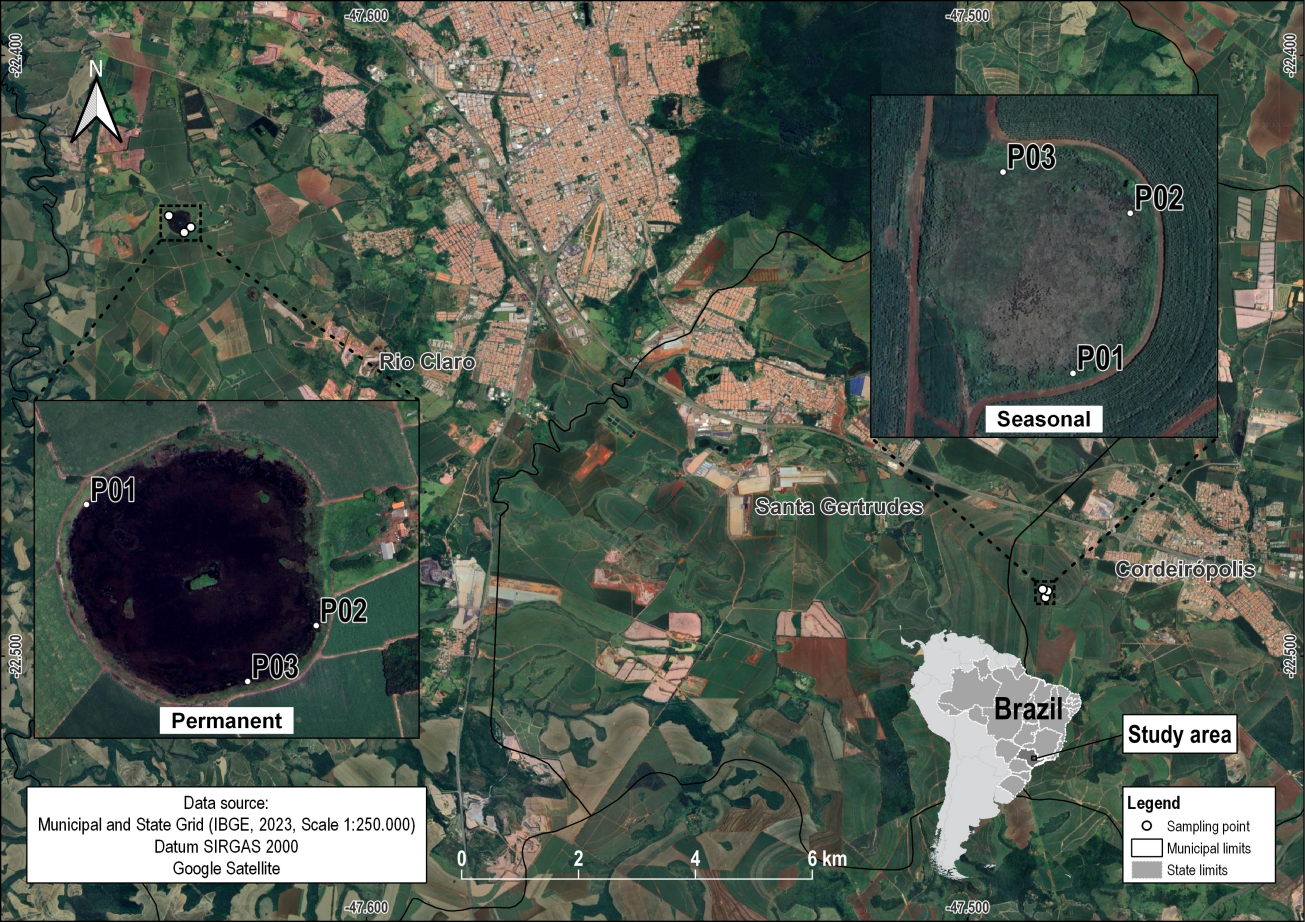
Map of the study area showing the location of both wetlands, sampling sites and surrounding features. Insets: close‐up views of each wetland.

The study area comprises two wetlands, one permanent and one seasonal, located in the municipality of Rio Claro and Cordeirópolis, in São Paulo State, Brazil (Figure 1), supported by claystones from the Corumbataí Formation overlaid by sandstones from the Rio Claro Formation and by diabases from the Serra Geral Formation, respectively.

The area was originally covered by *Cerrado* and forests (Barros et al. 2021); however, the intense process of deforestation and disorderly occupation in the basin led to the restriction of the remaining forest cover to sloping terrains and isolated stretches around waterbodies (Mori et al. 2016). Currently, the area is located in the rural area of the municipality, where there was an initial predominance of coffee cultivation, later replaced by sugarcane cultivation (Barros et al. 2021). Sugarcane cultivation occupies soils with distinct physicochemical characteristics and a flat to mountainous relief, which generates great concern about the environmental behaviour of pollutants in this basin. The Corumbataí River is in the process of eutrophication, and the metals zinc, lead, cadmium and nickel are frequently detected in the basin (Maranho 2012). The area is characterised by a relief system of broad to medium hills and elongated hills and ridges, at an altitude of 600–650 m (Bistrichi and Almeida 1981). The climate is classified as high‐altitude tropical, with an average annual temperature of 21.6°C and an average annual rainfall of 1366.8 mm. The highest rainfall rates are observed in the summer months (Cepagri 2020).

In the permanent wetland, standing water remained 10–30 cm above the soil surface during the rainy season, and the water table receded to 10–20 cm below the surface during the dry season. In the seasonal wetland, water levels were 20–30 cm below the soil surface in the rainy season and dropped to more than 1 m below the surface during the dry season.

### 2.2 Collection and Storage of Samples

A total of 24 soil samples were analysed in this study. In each wetland, three points were sampled at the borders (six points in total). Soil samples were taken from the top 0–10 cm and 10–20 cm after removing the surface vegetation. Sampling was conducted in April 2023 (wet season) and early September 2023 (dry season). The wet‐season sampling occurred slightly after the peak rainy period because unusually high rainfall that year hindered access and prevented the execution of part of the planned monitoring activities. The soil samples were collected in airtight plastic bags properly sealed and identified, kept inside iceboxes until transportation to the laboratory and then were maintained at −80°C until DNA extraction.

Soil samples for physicochemical parameters (soil moisture, pH and Eh) and nutrient analyses (N, P and K) were collected independently from the DNA samples. For each sampling point, three subsamples were taken from the 0–20 cm layer, composited, and homogenised by quartering to obtain a representative sample. These composite samples were then processed according to the analytical procedures described below.

### 2.3 Sample Preparation

Soil physicochemical properties were determined as follows: soil organic carbon (SOC) was quantified by the Walkley‐Black method using potassium dichromate oxidation and titration, following the procedure described by FAO (2019). Soil organic matter (SOM) was estimated from the SOC content.

Soil moisture was measured gravimetrically after drying the samples at 105°C for 24 h. Soil pH was determined in a 1:2.5 (w/v) soil‐to‐water suspension, after shaking for 60 s and resting for 1 h, using a pH metre calibrated with standard buffer solutions (pH 4.0 and 7.0). Redox potential (Eh) was measured with a platinum electrode (YSI Pro4, Yellow Springs Instruments, USA) and corrected using Zobell's solution as reference. Total N was determined by the Kjeldahl method. Available P and potassium K were extracted using an ion‐exchange resin, with P quantified colorimetrically and K determined by atomic emission spectrophotometry (EMBRAPA 2017).

DNA was extracted from manually homogenised soil samples (soil mass used for DNA extraction: 234–272 mg) using a PowerSoil DNA Isolation Kit (Mobio Laboratories Inc., Carlsbad, CA, USA), following the manufacturer's protocol. No sieving or compositing procedures were applied. The quantity and quality of the DNA extracts were assessed using spectrophotometry (NanoDropTM). DNA quality was further evaluated by agarose gel electrophoresis (0.8% w/v) at a constant voltage of 3 V/cm in 1X TEB buffer, followed by staining with ethidium bromide and visualisation alongside a molecular marker (1 kb DNA Ladder RTU—KASVI). The DNA extracts were stored at −20°C. No negative extraction or library controls were included in this study. Subsequently, the samples were shipped for 16S rRNA gene amplification (V3‐V4 region), purification and sequencing at Novogene America (Sacramento, CA, USA), using their standard quality control workflow, which includes DNA quality assessment, removal of low‐quality reads, trimming of primers and barcodes, and chimera filtering.

Two dry‐season samples (one from each wetland type) failed the sequencing quality control and were excluded from the dataset, resulting in a total of 22 samples. The sequencing data generated in this study have been deposited in the NCBI repository under BioProject ID PRJNA1254781.

### 2.4 Raw Data Processing

The raw data obtained from sequencing were processed using QIIME2 version 2023.9, following the instructions described by Bolyen et al. (2019). The table of Amplicon Sequence Variants (ASVs) was generated using the DADA2 pipeline (Callahan et al. 2016), which includes the filtering and truncation of forward and reverse sequences to eliminate low‐quality regions, followed by merging of paired reads and chimera removal. For taxonomic identification, the SILVA database version 138 was used, applying a 97% cutoff threshold for taxonomic assignment (Quast et al. 2012). Shannon and Simpson diversity indices, as well as Chao1 and observed richness (observed ASVs), were calculated to assess the diversity and richness of the taxa present in the samples.

### 2.5 Metabolic Profile Analysis

Metabolic profiles were analysed using the Functional Annotation of Prokaryotic Taxa (FAPROTAX) program version 1.2.7 (Louca et al. 2016). This process involved associating the taxa identified through the SILVA database with potential ecological functions. Functional assignments were carried out by mapping the identified ASVs to functional categories in the FAPROTAX database, which includes approximately 80 documented ecological functions related to specific taxa of 16S rRNA gene. Each identified taxon was mapped to ecological functions such as fermentation, nitrogen fixation, among others, based on the predefined rules in FAPROTAX. This analysis is crucial to understanding the potential contribution of the identified microbes to the ecological processes of the studied ecosystem.

### 2.6 Figure Creation

All figures presented in this study were created using RStudio version 2024.04.1‐748, and the ggplot2 package, which is part of the R software environment (Version 4.2.1; RStudio Team 2024).

Rarefaction curves were created to illustrate the relationship between sequencing depth and the number of observed ASVs for each sample. For downstream alpha diversity analyses, the ASV table was rarefied to a common sequencing depth of 78,440 reads per sample to normalise sequencing effort and ensure comparability across samples. Alpha diversity metrics (Observed richness, Chao1, Shannon and Simpson) were calculated from this rarefied dataset.

For the statistical analyses, the microbial consortium data were grouped according to wetland type (permanent and seasonal), season (wet and dry) and depth (0–10 cm and 10–20 cm). The normality of the variables was assessed by Shapiro–Wilk test, while the differences between the means of comparing groups were assessed by paired *t*‐student test, independent *t*‐student test, or one‐way analysis of variance (ANOVA) and post hoc Tukey HSD. Shannon α‐diversity was compared across groups using one‐way ANOVA. For β‐diversity, abundance data were transformed using centered log‐ratio (CLR), and community dissimilarities were calculated using Euclidean distance in CLR. Differences in community composition were assessed through permutational multivariate analysis of variance (PERMANOVA), and the homogeneity of multivariate dispersions was evaluated using PERMDISP.

For multivariate analyses, relative abundance data were CLR transformed to account for the compositional nature of sequencing data, whereas untransformed relative abundances were used for taxonomic description and comparative purposes.

## 3 Results and Discussion

### 3.1 Microbial Diversity

The rarefaction curves and diversity indices, including Shannon and Simpson, can be found in the Supporting Information (Figures S1 and S2). The rarefaction curves have stabilised, indicating that sequencing depth was sufficient to capture most of the detectable ASV diversity within individual samples.

The permanent wetland showed consistently high Shannon diversity at both depths (Supporting Information, Table S1). At the surface layer (0–10 cm), Shannon values ranged from 6.93 to 7.27 across seasons, while in the 10–20 cm layer values ranged from 6.62 to 7.1. A similar pattern was observed for Chao1, with surface estimates ranging from 2360 to 3681 and deeper samples ranging from 2553 to 3295. Seasonal wetland exhibited similar average diversity. Shannon values ranged from 6.92 to 7.32 at the surface and 6.46 to 7.11 at 10–20 cm. Observed richness ranged from 2410 to 4465 at the surface and 2354 to 3507 at depth, while Chao1 ranged from 2378 to 4321 and 2354 to 3446, respectively.

Shannon diversity did not differ significantly between wetland types or seasons, indicating that overall community diversity was stable across these environmental gradients. Depth showed a significant effect, with lower diversity at 10–20 cm, suggesting a vertical structuring of microbial communities independent of hydrological regime or seasonal variability (Supporting Information, Figure S2; Tables S2 and S3). This pattern likely reflects higher inputs of organic matter at the surface, greater microbial activity, increased relative oxygen availability and a more heterogeneous set of micro‐habitats.

The 16S rRNA gene sequencing recovered both bacterial and archaeal ASVs, allowing the analysis of both domains. Community composition differed significantly across wetland type, season and depth for both domains. Archaea showed a moderate but significant response to wetland type, season and depth (PERMANOVA, *R* ^2^ = 0.38, *p* = 0.012). Bacterial communities exhibited a higher proportion of explained variation associated with these environmental gradients (*R* ^2^ = 0.42), although PERMDISP indicated differences in dispersion between wetland types (*p* = 0.045). This suggests that, for bacteria, both shifts in community centroids and differences in within‐group variability contributed to the observed compositional patterns. In contrast, archaeal community dispersion was homogeneous across all factors (PERMDISP, *p* > 0.05).

We identified 48 bacterial phyla across the two wetland types, seasons and depths. These results are in line with previous ones, which reported considerable differences between the soil microbial consortium of created wetlands with different hydrological regimes, highlighting the importance of the flooding regime on the microbial community (Ahn and Peralta 2009; Ligi et al. 2014).

**FIGURE 2:**
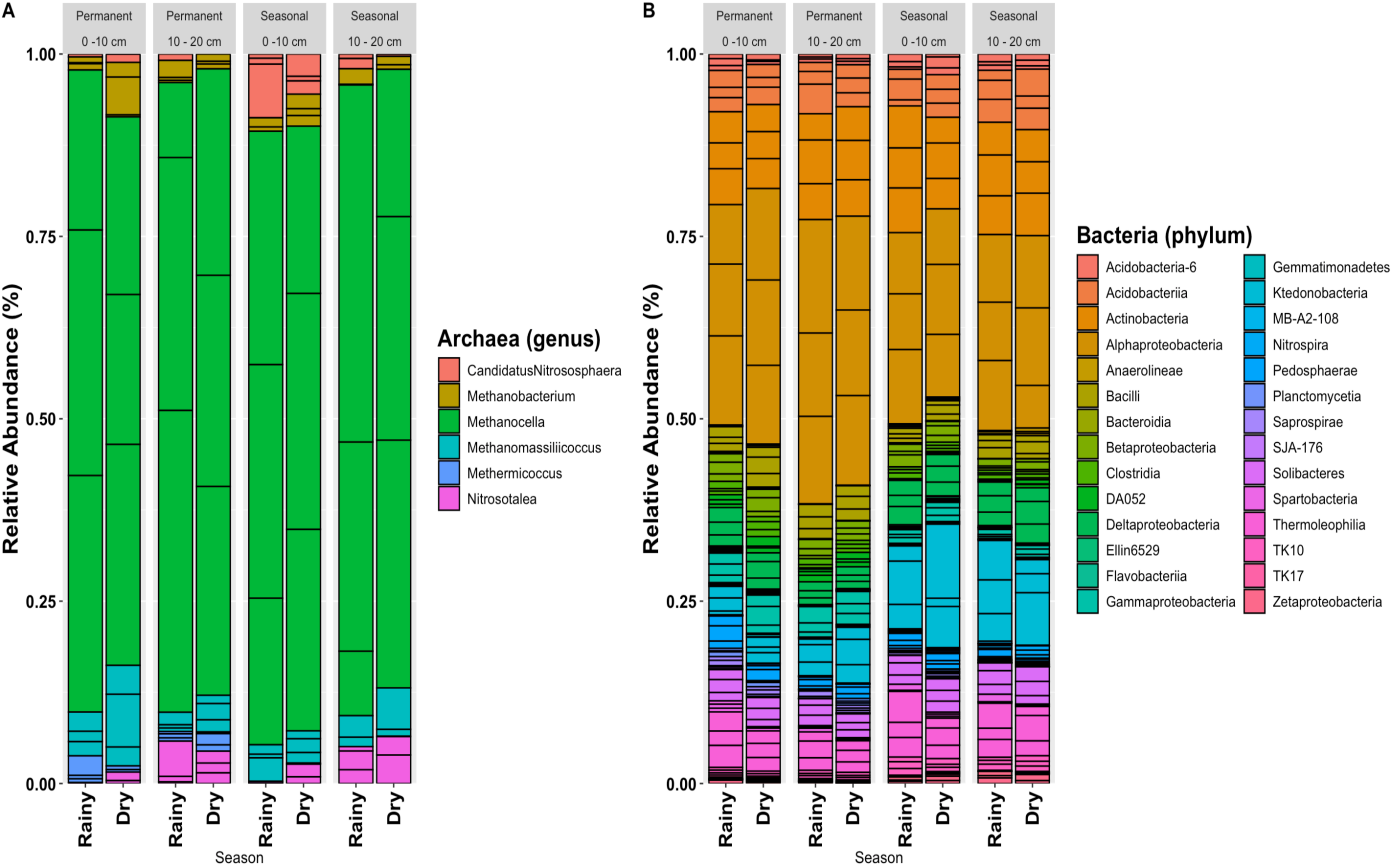
Stacked plot of the archaeal (A) and bacterial (B) diversity in the samples by wetland type, depth and season (*n* = 3).

In this study, 83% of the bacterial sequences consistently corresponded to six dominant phyla: *Acidobacteria*, *Actinobacteria*, *Bacteroidetes*, *Chloroflexi*, *Proteobacteria* and *Verrucomicrobia* (Figure 2).

This bacterial diversity corroborates findings from previous studies. For instance, Ligi et al. (2014) reported 56 bacterial phyla, with over 65% of sequences in each sample belonging to a similar core group of phyla, also indicating a predominance of a few taxa. Similarly, Peralta et al. (2013) found that five major bacterial groups (*Acidobacteria, Actinobacteria, Bacteroidetes, Firmicutes* and *Proteobacteria*) constituted more than 80% of sequences in various soil samples from natural and constructed wetlands in Virginia. Furthermore, Koloti et al. (2024) reported *Proteobacteria* and *Bacteroidetes* as the dominant microbial communities in diverse wetland ecosystems influenced by anthropogenic activities, while *Firmicutes* were prevalent in environments impacted by organic and heavy metal pollution. These emphasise that a few bacterial phyla dominate microbial communities across different wetland environments, indicating the potential functional importance of these core taxa in wetlands.

The dominant phyla detected, considering a minimum of 0.1% per sample, were *Proteobacteria* (ranged from 21.4% to 55.8% across the samples), *Acidobacteria* (3.8%–25.6%), *Actinobacteria* (1.9%–24.3%), *Bacteroidetes* (4.6%–13.2%), *Verrucomicrobia* (2.1%–6.8%), *Chloroflexi* (1.3%–4.7%), *Planctomycetes* (0.9%–3.1%), *Gemmatimonadetes* (0.4%–6.9%), WS3 (0.4%–2.3%), *Nitrospirae* (0.2%–6.5%), *Firmicutes* (0.1%–3.8%) and *Elusimicrobia* (0.1%–0.2%). The proportion of unclassified sequences varied from 3.1% to 14.8% in the studied soils. The taxonomic hierarchy from phylum to family, with relative abundances indicated for each taxon, is provided in Supporting Information S2.

**FIGURE 3:**
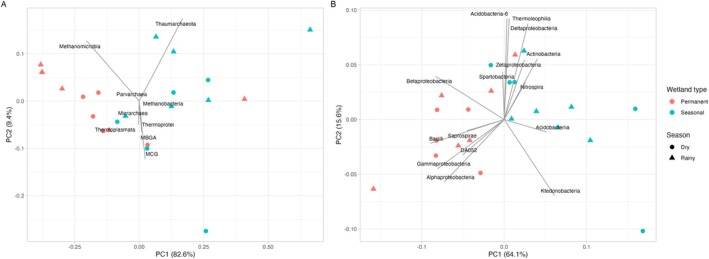
PCA comparing the Archaeal (A) and Bacterial (B) communities (Classes) based on CLR‐transformed relative abundance data at phylum level per wetland type and season.

When comparing the diversity of *Archaea* at the phylum level in the permanent and seasonal wetland, a clear separation between permanent and seasonal wetlands is observed, with a less pronounced distinction between the wet and dry seasons within these wetland types (Figure 3a).

In the seasonal wetland, there is a predominance of the phylum *Crenarchaeota*. In turn, the permanent wetlands are dominated by the phylum *Euryarchaeota*. At genus level (Supporting Information S2), the most abundant Archaea in the permanent wetland were *Methanocella* (rainy: 56%; dry: 51%), *Methanomassiliicoccus* (rainy: 3%; dry: 7%), *Methermicoccus* (rainy: 2%; dry: 1%), *Methanobacterium* (rainy: 2%; dry: 0%) and *Nitrosotalea* (rainy: 2%; dry: 2%), while in the seasonal wetland the most abundant genera were *Methanocella* (rainy: 32%; dry: 28%), *Methanomassiliicoccus* (rainy: 2%; dry: 2%), *Candidatus Nitrososphaera* (rainy: 2%; dry: 1%), *Nitrosotalea* (rainy: 1%; dry: 2%) and *Methanobacterium* (rainy: 1%; dry: 1%). At family level, *Methanocellaceae* was the most common across all the samples, depths and hydrological regime (16%–78%), being higher at the permanent wetland (ANOVA, *p* < 0.05), refer to the Supporting Information (Table S4) for a summary of these statistical outputs. This family comprises anaerobic microorganisms that use CO_2_ as an electron acceptor, generating CH_4_ and use H_2_ as an electron donor (Sakai et al. 2014). The microbial consortium from the two wetlands was markedly different from peat bogs at Orient, Maine, USA, in which at a similar depth (15 cm), another hydrogenotrophic group, *Methanoregulaceae* was the dominant family (5.33%–28.25%) (Arnold et al. 2023). Another important group in both hydrological regimes, with increased predominance in the seasonal wetland, was the *Nitrososphaeraceae* family. *Nitrososphaeraceae* is a strictly aerobic group that possesses the ability of performing chemolithoautotrophic growth by oxidising ammonia and fixing CO_2_ (Kerou and Schleper 2016).

When considering the diversity of phyla of *Bacteria* in the wetlands (Figure 3b) a distinct separation between the permanent and seasonal wetland, with a less prominent separation between the seasons, is also observed, similar to *Archaea*. The most abundant Bacteria phyla in the permanent wetland were *Proteobacteria* (rainy: 41%; dry: 43%), *Actinobacteria* (rainy: 19%; dry: 18%), *Acidobacteria* (rainy: 12%; dry: 11%), *Chloroflexi* (rainy: 6%; dry: 6%), *Firmicutes* (rainy: 5%; dry: 7%), *Verrucomicrobia* (rainy: 4%; dry: 3%), *Bacteroidetes* (rainy: 2%; dry: 2%) and *Planctomycetes* (rainy: 1%; dry: 1%). In the seasonal wetland the most abundant phyla were *Proteobacteria* (rainy: 32%; dry: 31%), *Actinobacteria* (rainy: 24%; dry: 19%), *Chloroflexi* (rainy: 14%; dry: 14%), *Acidobacteria* (rainy: 11%; dry: 13%), *Firmicutes* (rainy: 3%; dry: 3%), *Verrucomicrobia* (rainy: 3%; dry: 2%), AD3 (rainy: 1%; dry: 3%) and *Bacteroidetes* (rainy: 1%; dry: 1%). Proteobacteria has been widely reported as the dominating phylum in natural wetlands (Koloti et al. 2024; Zhou et al. 2024).

At class level, the most abundant Bacteria in the permanent wetland were *Alphaproteobacteria* (rainy: 30%; dry: 26%) and *Actinobacteria* (rainy: 12%; dry: 9%). In the seasonal wetland, the most abundant were *Alphaproteobacteria* (rainy: 22%; dry: 21%), *Actinobacteria* (rainy: 13%; dry: 11%) and Ktedonobacteria (rainy: 10%; dry: 11%).

These results corroborate findings previously reported of *Acidobacteria* and *Actinobacteria* being more abundant in seasonal wetland than in permanent ones (Ligi et al. 2014). In fact, the same phyla were reported by Ligi et al. (2014), with varying order of predominance. These authors reported *Proteobacteria* as the most dominant in all wetland soil samples, followed by either *Bacteroidetes* and *Acidobacteria* or *Acidobacteria* and *Actinobacteria*. Peralta et al. (2013) reported an increase in the *Acidobacteria* group in natural wetlands or created wetlands that already achieved an equilibrium. At the class level, *Alphaproteobacteria* (13%–41%), *Actinobacteria* (9%–16%), *Ktedonobacteria* (1%–26%), *Thermoleophilia* (3%–11%), *Acidobacteriia* (2%–11%), *Deltaproteobacteria* (2%–7%), *Solibacteres* (2%–5%), *Gammaproteobacteria* (1%–7%), *Bacilli* (1%–6%), *Betaproteobacteria* (1%–5%), *Clostridia* (0%–3%), TK10 (1%–2%), DA052 (0%–3%) and *Zetaproteobacteria* (0%–2%). Similar distribution was reported by Peralta et al. (2013) in both created and natural wetland soils in which *Alphaproteobacteria* was the dominant group, followed by *Delta‐* and *Betaproteobacteria*.

### 3.2 Metabolic Processes

**FIGURE 4:**
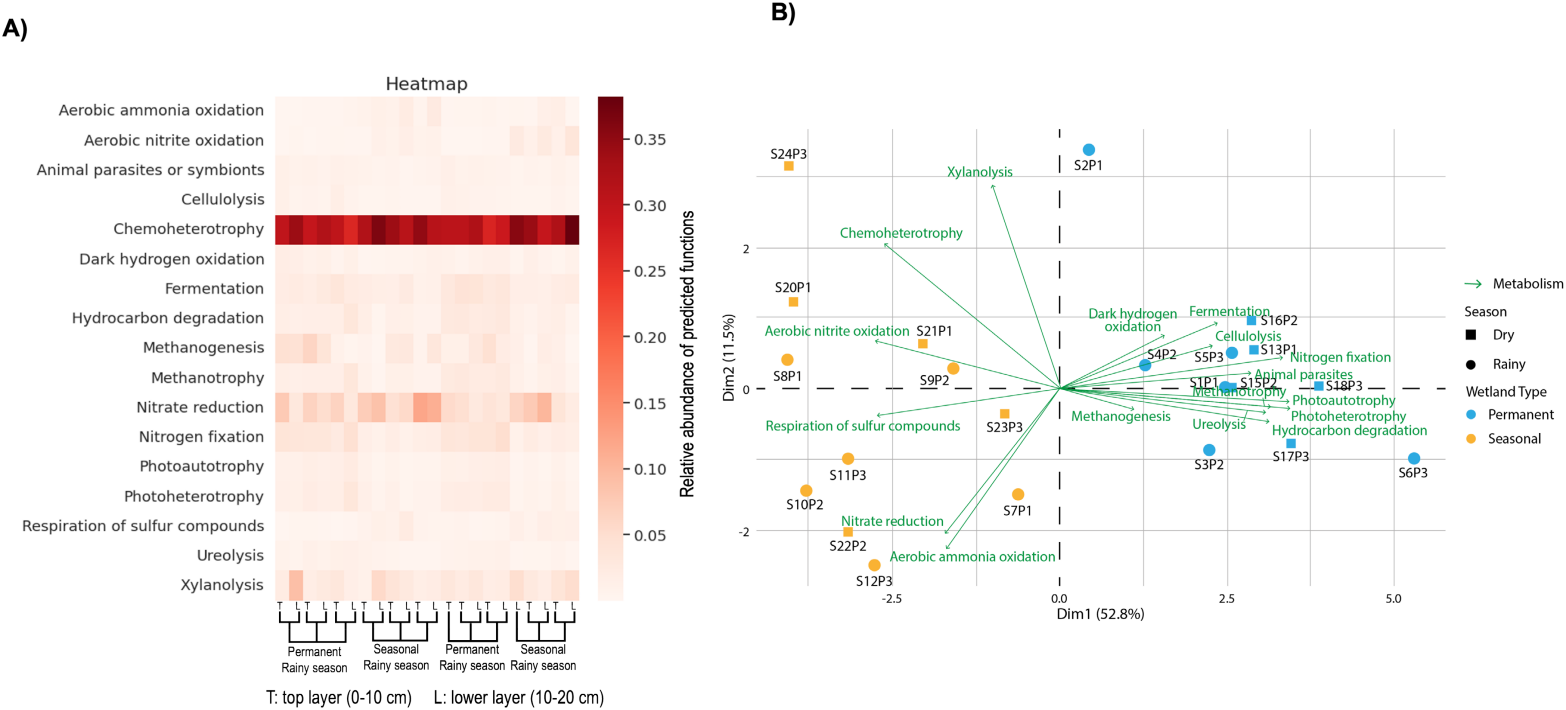
(A) Heatmap showing the main metabolic processes identified in samples from the permanent and seasonal wetlands, both seasons. (B) PCA comparing the main metabolic processes in the two wetland hydrological regimes.

When assessing the predominance of metabolic pathways in each wetland, it is possible to observe a higher prevalence of chemoheterotrophy in the seasonal wetland (Figure 4).

Chemoheterotrophy is the process of using organic carbon as an energy source (Alur 1999) and is a very ubiquitous metabolic route. Although it encompasses considerable metabolic diversity in terms of substrates and pathways, its high prevalence indicates that it alone is not a strong discriminator of metabolic potential between environments. Moreover, the phylum *Chloroflexi*, more prevalent in the seasonal wetland, can grow as aerobic chemoheterotrophs in the absence of light and is likely associated with the prevalence of chemoheterotrophy in the seasonal wetland (Thiel et al. 2019). Aerobic ammonia oxidation, nitrate reduction and sulphur compound respiration were also prominent metabolic pathways in the seasonal wetland. These pathways are strongly associated with the predominance of nitrifying microorganisms, including bacteria from the phylum *Nitrospirae*, which includes well‐characterised ammonia and nitrite oxidizers (Daims et al. 2015) and archaeal ammonia oxidizers such as *Nitrosotalea* and *Nitrososphaera* (Bei et al. 2024; Lehtovirta‐Morley et al. 2016). While some members of *Nitrospirae* may perform sulphate reduction in specific conditions (i.e., geothermal environments), the functional profiling of this dataset suggests that this pathway is predominantly driven by other lineages, such as *Desulfuromonas*, *Desulfovirga* and *Desulfovibrio*, which are well‐known sulphate‐reducing bacteria (Zhuang et al. 2024) and were ubiquitous across all samples, likely being the primary contributors to dissimilatory sulphate reduction. In turn, the metabolic processes prevalent in the permanent wetland included methanogenesis, fermentation, dark hydrogen oxidation, nitrogen fixation, photoautotrophy, ureolysis and hydrocarbon degradation. The predominance of methanogenesis, fermentation, dark hydrogen oxidation and ureolysis in the permanent wetland is expected due to the presence of anoxic hotspots, which favour anaerobic and specialised metabolic processes. The predominance of these diverse metabolic processes in the permanent wetlands suggests a more complex metabolic network compared to the seasonal wetland.

It is important to note that the taxonomic classification revealed that between 72.59% and 83.48% of the amplicon sequence variants (ASVs) remained unclassified at the genus level. This limitation may restrict the ability of the FAPROTAX approach to fully capture the metabolic potential of the microbial community. In addition, any functional inference based on 16S rRNA data provides only putative functional potential, since many metabolic traits are not strictly phylogenetically conserved (Djemiel et al. 2022). Therefore, the resulting functional profiles should be interpreted as conservative estimates. Nevertheless, the metabolic pathways identified were highly consistent with the environmental gradients and characteristics observed in the seasonal and permanent wetlands, reinforcing the ecological robustness of the functional inference despite the partial taxonomic resolution.

### 3.3 Environmental Drivers

The pH values in the soils did not differ between seasons for either the seasonal or permanent wetlands (paired *t*‐test, *p* > 0.05). Similarly, Eh showed no significant difference between seasons in both the seasonal and permanent wetlands (paired *t*‐test, *p* > 0.05). Soil moisture content also remained consistent across seasons for both wetland types (paired *t*‐test, *p* > 0.05). Although no significant seasonal differences in soil moisture were detected, the seasonal wetland experiences episodic flooding and prolonged saturation during the wet season, consistent with its classification based on hydrological regime rather than the measured surface moisture values. Because the soil moisture did not differ across the season not only for the permanent wetland, as expected, but also for the seasonal wetland, it can be inferred that the microbiota herein assessed is not subject to dry‐wet cycles, which are highly stressful for microorganisms (Fierer et al. 2003). However, when comparing the two wetland types, significant differences were observed in both Eh and soil moisture, with the permanent wetland showing lower Eh and higher soil moisture compared to the seasonal wetland (independent *t*‐test, *p* < 0.01).

The seasonal and spatial variations in nutrient concentrations between the two wetland types were statistically evaluated and compared with microbial diversity patterns. The concentrations of total N, available P and K are presented on Table 1. Based on these data, N levels did not vary significantly between seasons in either wetland (paired *t*‐test, *p* > 0.05) or between the two wetland types (independent *t*‐test, *p* > 0.05). Phosphorus levels showed a significant difference between seasons only in the permanent wetland (paired *t*‐test, *p* < 0.05), but not in the seasonal wetland (paired *t*‐test, *p* > 0.05). Additionally, P levels were significantly different between the two wetland types (independent *t*‐test, *p* = *p* < 0.05), being higher in the seasonal wetland. Potassium levels did not differ significantly between seasons in either wetland type (paired *t*‐test, *p* > 0.05) or between the wetland types (independent *t*‐test, *p* > 0.05). A summary of the statistical outputs for pH, Eh, TOC, soil moisture and macronutrients are available in the Supporting Information (Table S5).

**TABLE 1:** Concentration of SOC and NPK in soil samples (mean ± SD, *n* = 2) at 0–20 cm.

Wetland type	Season	Sampling point	SOC (%)—soil	N (mg Kg^−1^)	P (mg Kg^−1^)	K (mg Kg^−1^)
Permanent	Rainy	P1	2.3 ± 0.2	1897 ± 69	16 ± 1	1756 ± 194
		P2	5.1 ± 0.1	4235 ± 178	19.3 ± 0.4	1572 ± 100
		P3	1.17 ± 0.01	1043 ± 139	12 ± 1	864 ± 44
	Dry	P1	2.09 ± 0.01	2104 ± 5	11 ± 1	1105 ± 180
		P2	3.75 ± 0.04	3854 ± 153	14 ± 1	1073 ± 163
		P3	2.5 ± 0.1	2580 ± 193	8.8 ± 0.1	1239 ± 547
Seasonal	Rainy	P1	2.64 ± 0.07	2783 ± 94	28.0 ± 0.9	1069 ± 473
		P2	2.6 ± 0.1	2723 ± 49	37.2 ± 0.1	1069 ± 3
		P3	3.7 ± 0.3	3192 ± 20	70.5 ± 0.6	5108 ± 3
	Dry	P1	2.31 ± 0.04	2352 ± 188	25.5 ± 0.2	1157 ± 271
		P2	2.3 ± 0.1	2282 ± 198	40.2 ± 0.3	6127 ± 144
		P3	2.01 ± 0.03	2254 ± 346	23.3 ± 0.1	1720 ± 55

#### 3.3.1 Bacteria

The two wetlands investigated in this study exhibited fundamentally distinct ecological structures shaped by different sets of environmental constraints. In the permanent wetland, microbial community distribution was consistent with long‐term saturation and chronic nutrient limitation (Table 2). No significant correlations were observed between pH and bacterial taxa in the permanent wetland. In contrast, in the seasonal wetland, pH emerged as a major structuring factor, particularly in the deeper layer. Several bacterial classes exhibited strong negative correlations with pH, including *Acidobacteriia*, *Anaerolineae, Actinobacteria* and multiple *Chloroflexi*‐related lineages, corroborating previous reports that link these groups to acidic soil environments (Xia et al. 2020; Zhou et al. 2024). Positive correlations were also found between pH and Flavobacteria and Saprospirae, frequently reported to exhibit neutrophilic or alkaliphilic optima (Zhou et al. 2024). Across the seasonal wetland, filamentous bacterial lineages consistently responded to acidic and stressful conditions. Filamentous morphology has been proposed as an adaptive strategy under environmental stress, conferring advantages such as enhanced tolerance to physicochemical gradients, improved surface colonisation and the ability to span heterogeneous microsites (Karasz et al. 2022). The recurrent association of filamentous taxa, including Actinobacteria and *Chloroflexi*‐related groups, with low pH and reduced conditions in this study suggests that filamentation may represent a competitive advantage in microenvironments where diffusion is limited and physicochemical conditions fluctuate rapidly.

**TABLE 2:** Pearson correlations between bacterial taxa (CLR‐transformed abundances) and selected soil biogeochemical parameters.

Wetland	Environmental drivers	Variable	Bacterium	*R* ^2^	*p*	Depth	Description	References
Permanent	Physicochemical properties	Eh	Gemmatimonadetes	−0.80	< 0.05	0–10 cm	Aerobic drought‐tolerant	(Kamagata 2015; Mujakić et al. 2023)
			Betaproteobacteria	−0.77	< 0.05		Facultative anaerobes/denitrifiers	(Boden 2024)
	Soil macronutrients	P	Thermoleophilia	0.78	< 0.05	10–20 cm	Filamentous oligotrophic	(Hu et al. 2019)
			Pedosphaerae	0.70	< 0.05		Oligotrophic	(Bergmann et al. 2011)
			Actinobacteria	0.68	< 0.05		Filamentous	(Karasz et al. 2022)
		K	Zetaproteobacteria	0.68	< 0.05	10–20 cm	Fe‐oxidizers	(McAllister et al. 2019)
			Solibacteres	0.78	< 0.05		Oligotrophic	(Xia et al. 2020)
			DA052	0.72	< 0.05		Acidobacteria‐associated candidate clade	—
			Actinobacteria	0.68	< 0.05		Filamentous	(Karasz et al. 2022)
			Saprospiria	−0.72	< 0.05		Copiotrophic degraders	(Liu et al. 2022)
Seasonal	Physicochemical properties	pH	SJA‐176	−0.91	< 0.01	0–10 cm	Filamentous Chloroflexi	(Zhou et al. 2024)
			Ktedonobacteria	−0.80	< 0.01		Filamentous Chloroflexi	(Yabe et al. 2017; Zhou et al. 2024)
			Ellin6529	0.96	< 0.005		Cloroflexi‐associated candidate clade	—
			Solibacteres	−0.81	< 0.05	10–20 cm	Oligotrophic	(Xia et al. 2020)
			Acidobacteriia	−0.94	< 0.01		Oligotrophic	(Xia et al. 2020; Zhou et al. 2024)
			Actinobacteria	−0.99	< 0.005		Filamentous	(Karasz et al. 2022)
			Alphaproteobacteria	−0.80	< 0.05		Oligotrophic phototrophs (Rhodospirillales)	(Hölzl and Dörmann 2007)
			Deltaproteobacteria	−0.87	< 0.01		Anaerobic/facultative	—
			Anaerolineae	−0.82	< 0.05		Strict anaerobes	(Narihiro et al. 2012)
			Saprospirae	0.91	< 0.01		Copiotrophic degraders	(Liu et al. 2022)
			Flavobacteria	0.89	< 0.01		Copiotrophic degraders	(Liu et al. 2022)
			MB.A2.108	0.71	< 0.05		Candidate clade	—
	
		Eh	Thermoleophilia	−0.90	< 0.01	0–10 cm	Filamentous oligotrophic	(Hu et al. 2019)
			Nitrospira	0.82	< 0.05		Nitrifiers	(Chisholm et al. 2023; Ludwig et al. 2015)
			Gammaproteobacteria	0.84	< 0.05		Aerobic/facultative	(Ludwig et al. 2015)
		Soil moisture	Gemmatimonadetes	0.71	< 0.05	0–10 cm	Aerobic drought‐tolerant	(Kamagata 2015; Mujakić et al. 2023)
			TK17	−0.68	< 0.05		Filamentous Chloroflexi	—
			Zetaproteobacteria	0.77	< 0.05	10–20 cm	Fe‐oxidizers	(McAllister et al. 2019)
			Betaproteobacteria	−0.70	< 0.05		Facultative anaerobes/denitrifiers	(Boden 2024)
		SOC	Bacteroidia	0.82	< 0.05	10–20 cm	Copiotrophic degraders	(Liu et al. 2022)
			Planctomycetia	−0.83	< 0.05		Slow‐growing oligotrophs	—
	Nitrospira		−0.78	< 0.05	Nitrifiers		(Chisholm et al. 2023)
	Soil macronutrients	N	Thermoleophilia	0.74	< 0.05	0–10 cm	Filamentous oligotrophic	(Hu et al. 2019)
			Pedosphaerae	0.66	< 0.05	10–20 cm	Oligotrophic	(Bergmann et al. 2011)
			Nitrospira	−0.68	< 0.05		Nitrifiers	(Chisholm et al. 2023)
		P	Planctomycetia	0.92	< 0.05	10–20 cm	Slow‐growing oligotrophs	—
			Flavobacteria	0.74	< 0.05		Copiotrophic degraders	(Liu et al. 2022)
			Bacteroidia	0.76	< 0.05		Copiotrophic degraders	(Liu et al. 2022)
			TK10	−0.83	< 0.05		Filamentous Chloroflexi	—
			Nitrospira	−0.68	< 0.05		Nitrifiers	(Chisholm et al. 2023)
			Clostridia	−0.68	< 0.05		Strict anaerobes	—
			Bacilli	−0.68	< 0.05		Aerobic/facultative	—
			Anaerolineae	−0.67	< 0.05		Strict anaerobes	(Narihiro et al. 2012)
Acidobacteriia			−0.69	< 0.05	Oligotrophic		(Eichorst et al. 2007)
	
		K	Ellin6529	0.81	< 0.05	0–10 cm	Cloroflexi‐associated candidate clade	—
			SJA‐176	−0.95	< 0.01		Filamentous Chloroflexi	(Zhou et al. 2024)
			Ktedonobacteria	−0.76	< 0.05		Filamentous Chloroflexi	(Zhou et al. 2024)
			Saprospiria	0.97	< 0.005	10–20 cm	Copiotrophic degraders	(Liu et al. 2022)
			Flavobacteria	0.91	< 0.01		Copiotrophic degraders	(Liu et al. 2022)
			TK10	−0.84	< 0.05		Filamentous Chloroflexi	—
			Ktedonobacteria	−0.66	< 0.05		Filamentous Chloroflexi	(Zhou et al. 2024)
			DA052	−0.70	< 0.05		Acidobacteria‐associated candidate clade	—
			Alphaproteobacteria	−0.72	< 0.05		Oligotrophic phototrophs (Rhodospirillales)	(Hölzl and Dörmann 2007)
			Actinobacteria	−0.92	< 0.01		Filamentous	(Karasz et al. 2022)
Acidobacteriia	−0.89		< 0.01	Oligotrophic	(Eichorst et al. 2007)	

In both permanent and seasonal wetlands, correlations with Eh were restricted to a small number of bacterial taxa. In addition, the absence of significant correlations with soil moisture in the permanent wetland is consistent with moisture not acting as a driver in this system, possibly as saturation remained nearly constant throughout the year.

Despite the central role of carbon in microbial metabolism, relatively few significant correlations were observed between bacterial taxa and soil carbon. This apparent contradiction is likely explained by the fact that the measured variable represents SOC, which does not necessarily reflect substrate availability. Consequently, the limited number of correlations does not imply a minor role of carbon. Where correlations were observed, they were concentrated in the deeper layer of the seasonal wetland, where carbon‐rich microsites favoured copiotrophic taxa such as *Bacteroidia*, while disadvantaging slower‐growing groups such as *Planctomycetia* and nitrifiers (Chisholm et al. 2023). These patterns likely reflect competitive interactions rather than direct carbon limitation.

In the permanent wetland, slight increases in P and K were associated with oligotrophic taxa such as *Thermoleophilia*, *Pedosphaerae*, *Solibacteres* and DA052, suggesting that small variations in nutrient availability may alleviate chronic nutrient limitation in highly weathered tropical soils (Cui et al. 2024). In such environments, both P and K function as relevant components of the limiting nutrient matrix, and oligotrophic lineages with high‐affinity uptake systems and enzymatic mechanisms adapted to low nutrient supply appear to respond more consistently to these small variations. In more fertile soils, they lose space to fast‐growing copiotrophic groups (Finn et al. 2017). The negative correlation between K and the copiotrophic class *Saprospiria* suggests that copiotrophic taxa did not benefit from these subtle nutrient gradients. A similar pattern has been reported in estuarine systems, where *Saprospirae* became less competitive as nutrient concentrations declined following the dilution of nutrient‐rich freshwater into oligotrophic seawater, a response attributed to their reduced competitiveness under low‐nutrient conditions (Navarro et al. 2023).

In the seasonal wetland, copiotrophic groups responded positively to higher P and K availability in the deeper layer, whereas oligotrophic and stress‐tolerant taxa, such as *Ktedonobacteria* (Yabe et al. 2017) were negatively correlated with these nutrients. Copiotrophic groups with faster growth and the ability to exploit organic‐rich or nutrient‐rich substrates, such as Flavobacteria, *Saprospirae* and *Bacteroidia*, showed positive correlations with P and K. These taxa comprise efficient degraders, which respond rapidly to higher availability of nutrient input (Liu et al. 2022). Futhermore, the recurrent negative correlation of *Acidobacteriia*, DA052 and TK10 with P and K is consistent with these slow‐growing organisms having a competitive advantage in environments with low nutrient availability (Eichorst et al. 2007). *Alphaproteobacteria* represented majorly by anoxygenic photosynthetic *Rhodospirillales* (5%–9%) (Hölzl and Dörmann 2007), also slow‐growing and oligotrophic, showed a negative correlation with K as well.

Overall, the results support the existence of two contrasting ecological regimes. The permanent wetland is structured primarily by redox stability and chronic nutrient limitation, likely favouring slow‐growing oligotrophic lineages. In contrast, in the seasonal wetland, pH and nutrient gradients interact to shape microbial distributions.

#### 3.3.2 Archaea

Archaeal community patterns suggest a strong dominance of hydrogenotrophic methanogenesis in both wetlands, driven by the high relative abundance of *Methanocella*, which accounts for 16%–46% of the archaeal community (mean relative abundance = 28%). Significant correlations were only observed for archaeal taxa in the seasonal wetland (Table 3), and their responses were particularly pronounced in the upper layer, where SOC and nutrient availability strongly structured community composition.

**TABLE 3:** Pearson correlations between archaeal taxa (CLR‐transformed abundances) and selected soil biogeochemical parameters.

Wetland	Environmental drivers	Variable	Archaeal taxon	*R* ^2^	*p*	Depth	Description	References
Seasonal	Physicochemical properties	SOC	*Methermicoccus*	0.86	< 0.01	0–10 cm	Methylotrophic methanogen	(Lever and Teske 2015)
			*Methanomassiliicoccus*	0.77	< 0.05		Methylotrophic methanogen	(Nkamga and Drancourt 2016)
			*Methanocella*	0.86	< 0.01		Hydrogenotrophic methanogen	(Boone 2015; Sakai and Imachi 2016)
			*Methanobacterium*	−0.86	< 0.01		Hydrogenotrophic methanogen	(Boone 2015)
			*Nitrosotalea*	−0.74	< 0.05		Ammonia‐oxidising archaea	(Prosser and Nicol 2016)
		pH	*Methanocella*	−0.66	< 0.05	10–20 cm	Hydrogenotrophic methanogen	(Sakai and Imachi 2016)
	Soil macronutrients	N	*Nitrosotalea*	−0.75	< 0.05	0–10 cm	Ammonia‐oxidising archaea	(Prosser and Nicol 2016)
			*Methermicoccus*	0.68	< 0.05		Methylotrophic methanogen	(Lever and Teske 2015)
			*Methanocella*	0.66	< 0.05		Hydrogenotrophic methanogen	(Sakai and Imachi 2016)
		P	*Methermicoccus*	−0.71	< 0.05	10–20 cm	Methylotrophic methanogen	(Lever and Teske 2015)
		K	*Methermicoccus*	−0.67	< 0.05	10–20 cm	Methylotrophic methanogen	(Lever and Teske 2015)

*Nitrosotalea* was negatively correlated with SOC. Ammonia‐oxidising archaea are disfavored in the strongly reducing environments, such as carbon‐rich sites that support methanogenesis (Prosser and Nicol 2016). Nutrient availability also appeared to shape archaeal community patterns in the seasonal wetland. Nitrogen correlated negatively with *Nitrosotalea* but positively with *Methermicoccus* and *Methanocella*. Nitrogen was also negatively correlated with the other nitrifier, *Nitrospirae*. Total N accumulates largely in more reduced, carbon‐rich sites (TC × TN in the seasonal wetland: *R* ^2^ = 0.91; *p* < 0.05), where oxygen is scarce and nitrification is strongly constrained. In such zones, N remains predominantly in organic or ammonium forms, methanogenic and other anaerobic processes prevail, as indicated by the positive correlations with the methanogens *Methermicoccus* and *Methanocella*, both also correlated with SOC, and nitrifying taxa are effectively excluded.

It is important to note that correlation analyses in microbiome studies are constrained by factors such as the compositional nature of sequencing data, indirect associations and environmental drivers that were not included in the analysis. These limitations reduce their ability to represent true ecological interactions and reinforce the need for cautious interpretation (Carr et al. 2019).

## 4 Conclusion

We identified 48 bacterial phyla across the two wetland types, seasons and depths, and 83% of the bacterial sequences consistently corresponded to six dominant phyla: *Proteobacteria, Acidobacteria*, *Actinobacteria*, *Bacteroidetes*, *Verrucomicrobia* and *Chloroflexi*, corroborating previous studies and highlighting the fundamental role of this core group in wetland ecosystems. In the seasonal wetland, there is a predominance of the phyla *Chloroflexi, Nitrospirae*, *Actinobacteria* and *Acidobacteria*. In turn, the permanent wetland shows a prevalence of the phyla *Planctomycetes*, *Bacteroidetes*, *Proteobacteria* and *Firmicutes*. Regarding *Archaea diversity*, there is a predominance of the phylum *Crenarchaeota* in the seasonal wetland and *Euryarchaeota* in the permanent wetland. At family level, *Methanocellaceae* was the most common across all the samples, depths and hydrological regime (16%–78%), being higher at the permanent wetland. Overall, microbial communities exhibited marked compositional shifts across wetland type, season and depth, while maintaining stable alpha diversity. The decoupling between beta and alpha diversity suggests that changes in hydrological regime, seasonality and depth lead to a reorganisation of microbial assemblages without affecting overall community diversity. The consistent decrease in diversity at greater depth highlights vertical stratification as a fundamental organising factor of wetland microbial communities, overriding seasonal and hydrological influences.

Aerobic ammonia oxidation, nitrate reduction and respiration of sulphur compounds were putative metabolic pathways prevailing in the seasonal wetland. In turn, putative metabolic processes prevalent in the permanent wetland included methanogenesis, fermentation, dark hydrogen oxidation, nitrogen fixation, photoautotrophy, ureolysis and hydrocarbon degradation.

We interpreted the microbial patterns in light of the soil physicochemical properties, soil NPK content and inorganic electron acceptors to better distinguish the two wetland types; however, it is important to bear in mind that these interpretations are derived from correlation analyses and therefore represent a conceptual ecological model rather than direct evidence of mechanistic interactions. Within these constraints, the permanent wetland was characterised by sparse correlation patterns, consistent with a system primarily structured by long‐term saturation, redox stability and chronic nutrient limitation. In contrast, the seasonal wetland showed more numerous and diverse correlations, indicating a stronger influence of pH variability, nutrient gradients and SOC on microbial community structure.

## Author Contributions

**Karen Luko‐Sulato:** conceptualization, data curation, formal analysis, funding acquisition, investigation, methodology, project administration, resources, validation, visualization, writing – original draft. **Everton Tiago Sulato:** data curation, formal analysis, investigation, validation, visualization, writing – original draft. **Jorge R. Osman:** formal analysis, validation, visualization, writing – original draft. **Pedro Nolasco‐Jiménez:** formal analysis, visualization. **Daniela Morales:** formal analysis, visualization. **Graziela Silva Rezende:** investigation. **Cassy Anne Rodrigues:** writing – review and editing. **Sandra imaculada maintinguer:** writing – review and editing. **Anderson Ferreira da Cunha:** investigation, resources, supervision, validation, visualization, writing – original draft. **Vania Rosolen:** conceptualization, funding acquisition, investigation, methodology, project administration, resources, supervision, validation, visualization, writing – original draft.

## Funding

This work was supported by Fundação de Amparo à Pesquisa do Estado de São Paulo, Processo no. 2021/06332‐1, 2023/15396‐9.

## Conflicts of Interest

The authors declare no conflicts of interest.

## Supporting information


**Data S1:** Supporting Information 1.

**Data S2:** Supporting Information 2.

## Data Availability

The data that support the findings of this study are openly available in NCBI repository at https://www.ncbi.nlm.nih.gov/, reference number BioProject ID PRJNA1254781.
