# Supplementary material for: Soil Microbiome of Tropical Seasonal and Permanent Small Wetlands
Source: Environ Microbiol Rep. 2026 Mar 30;18(2):e70306. doi: 10.1111/1758-2229.70306 (PMC13053137; doi:10.1111/1758-2229.70306)
Supplement: Supplementary file 1 — Data S1: Supporting Information 1. [file EMI4-18-e70306-s001.docx]

**SOIL MICROBIOME OF TROPICAL SEASONAL AND PERMANENT SMALL WETLANDS**

Karen Luko-Sulato^1,6^, Everton Tiago Sulato^1^, Jorge R. Osman ^2,3^, Pedro Nolasco-Jiménez^3^, Daniela Morales^2^, Graziela Silva Rezende^4^, Cassy Anne Rodrigues^5^, Sandra Imaculada Maintinguer^6^, Anderson Ferreira da Cunha^4^, Vania Rosolen^7^

^1^Departamento de Geologia, Instituto de Geociências e Ciências Exatas (IGCE), Universidade Estadual Paulista (UNESP), Rio Claro, São Paulo, Brasil

^2^Instituto de Geología Económica Aplicada (GEA), Universidad de Concepción, Concepción, Chile.

^3^Instituto de Recursos Naturales y Agrobiología de Sevilla (IRNAS-CSIC), Av. Reina Mercedes 10, 41012 Sevilla, España

^4^Departamento de Genética e Evolução, Universidade Federal de São Carlos, São Carlos, São Paulo, Brasil

^5^Lab. de Ecologia Vegetal, Universidade Estadual Paulista (UNESP), Instituto de Biociências, Avenida 24A, 1515, CEP: 13506-900, Rio Claro, Brasil

^6^Instituto de Pesquisa em Bioenergia (IPBEN), Rua 10, 2527, Santana, Rio Claro, SP, 13500-230, Brasil

^7^Departamento de Engenharia Ambiental, Instituto de Geociências e Ciências Exatas (IGCE), Universidade Estadual Paulista (UNESP), Rio Claro, São Paulo, Brasil

*****Corresponding author

E-mail address: karen.luko@unesp.br


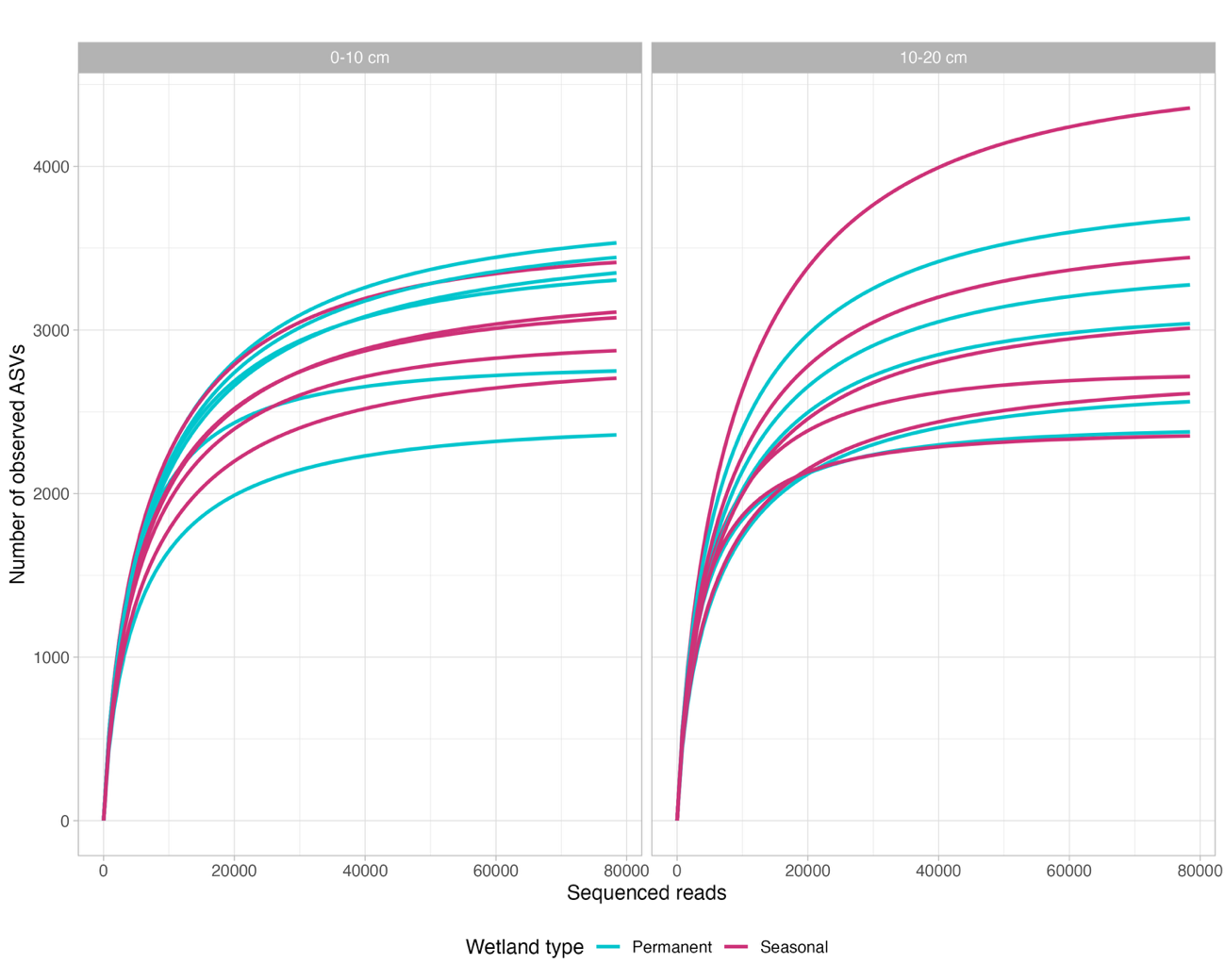


SF1. Rarefaction curves showing the number of observed ASVs as a function of sequencing effort for permanent and seasonal wetlands at 0-10 cm and 10- 20 cm depth.


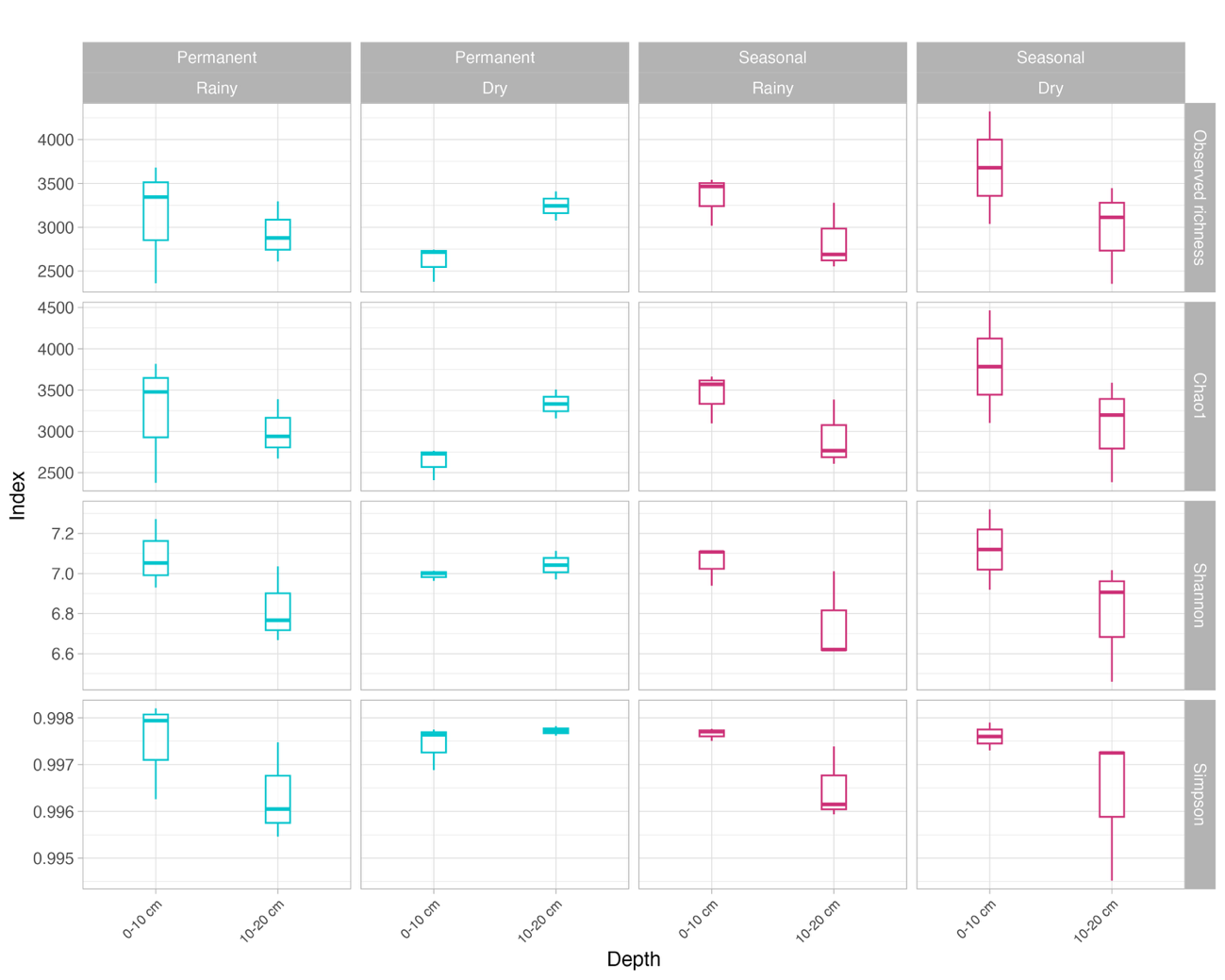


SF2. Boxplots displaying the distribution of rarefied alpha diversity indexes at different depths (0-10 cm and 10-20 cm) for each wetland type (Permanent and Seasonal).

| TS1: Alpha diversity comparing wetland type, depth and sampling season. Results are represented as range, mean ± standart deviation | | | | | | |
| --- | --- | --- | --- | --- | --- | --- |
| **Wetland type** | **Sampling season** | **Depth (cm)** | **Shannon** | **Simpson** | **Chao1** | **Observed richness** |
| Permanent | Rainy | 0-10 | 6.93 - 7.27;  7.1 ± 0.2 | 0.996 - 0.998; 0.998 ± 0.001 | 2360 - 3681;  3344 ± 686 | 2378 - 3817;  3478 ± 752 |
|  |  | 10-20 | 6.67 - 7.04;  6.8 ± 0.2 | 0.995 - 0.997; 0.996 ± 0.001 | 2609 - 3295;  2878 ± 346 | 2673 - 3389;  2940 ± 362 |
|  | Dry | 0-10 | 6.94 - 7.11;  7.1 ± 0.1 | 0.998 - 0.998; 0.998 ± 0.001 | 3017 - 3542;  3465 ± 284 | 3097 - 3663;  3570 ± 304 |
|  |  | 10-20 | 6.62 - 7.01;  6.6 ± 0.2 | 0.996 - 0.997; 0.996 ± 0.001 | 2553 - 3279;  2690 ± 386 | 2609 - 3386;  2767 ± 410 |
| Seasonal | Rainy | 0-10 | 6.96 - 7.01; 7.00 ± 0.03 | 0.997 - 0.998; 0.998 ± 0.001 | 2378 - 2745;  2715 ± 204 | 2410 - 2765;  2729 ± 195 |
|  |  | 10-20 | 6.97 - 7.11;  7.0 ± 0.1 | 0.998 - 0.998; 0.998 ± 0.001 | 3078 - 3409; 3243 ± 234 | 3157 - 3507;  3332 ± 248 |
|  | Dry | 0-10 | 6.92 - 7.32;  7.1 ± 0.3 | 0.997 - 0.998; 0.998 ± 0.001 | 3037 - 4321;  3679 ± 908 | 3104 - 4465; 3784 ± 962 |
|  |  | 10-20 | 6.46 - 7.02;  6.9 ± 0.3 | 0.995 - 0.997; 0.997 ± 0.002 | 2354 - 3446; 3113 ± 560 | 2354 - 3446;  3198 ± 572 |

| TS 2 – ANOVA Results for Alpha Diversity Indices | | | | | |
| --- | --- | --- | --- | --- | --- |
| Index | Factor | Df | F value | p-value | Interpretation |
| Shannon | Wetland | 1 | 0.747 | 0.3988 | ns |
| Shannon | Season | 1 | 0.259 | 0.6172 | ns |
| Shannon | Depth | 1 | 7.346 | 0.0143 | * |
| Simpson | Wetland | 1 | 0.340 | 0.5671 | ns |
| Simpson | Season | 1 | 0.306 | 0.5872 | ns |
| Simpson | Depth | 1 | 5.528 | 0.0303 | * |
| Observed richness | Wetland | 1 | 0.944 | 0.344 | ns |
| Observed richness | Season | 1 | 0.000 | 0.999 | ns |
| Observed richness | Depth | 1 | 0.786 | 0.387 | ns |
| Chao1 | Wetland | 1 | 1.014 | 0.327 | ns |
| Chao1 | Season | 1 | 0.005 | 0.945 | ns |
| Chao1 | Depth | 1 | 0.680 | 0.421 | ns |

| TS 3 – Tukey HSD Results for Alpha Diversity Indices | | | | |
| --- | --- | --- | --- | --- |
| Index | Comparison | Difference | 95% CI (lwr–upr) | p adj |
| Shannon | Wetland: Seasonal–Permanent | -0.0692 | -0.2374 to 0.0990 | 0.3988 |
| Shannon | Season: Dry–Rainy | 0.0409 | -0.1280 to 0.2098 | 0.6172 |
| Shannon | Depth: 10–20 – 0–10 cm | -0.2161 | -0.3843 to -0.0479 | 0.0147 |
| Simpson | Wetland: Seasonal–Permanent | -0.000219 | -0.001010 to 0.000571 | 0.5671 |
| Simpson | Season: Dry–Rainy | 0.000209 | -0.000585 to 0.001002 | 0.5872 |
| Simpson | Depth: 10–20 – 0–10 cm | -0.000881 | -0.001671 to -0.000091 | 0.0309 |
| Observed richness | Wetland: Seasonal–Permanent | 211.36 | -245.64 to 668.36 | 0.3441 |
| Observed richness | Season: Dry–Rainy | 0.18 | -458.72 to 459.08 | 0.9993 |
| Observed richness | Depth: 10–20 – 0–10 cm | -192.03 | -649.03 to 264.97 | 0.3890 |
| Chao1 | Wetland: Seasonal–Permanent | 235.41 | -255.78 to 726.60 | 0.3273 |
| Chao1 | Season: Dry–Rainy | -16.54 | -509.77 to 476.70 | 0.9446 |
| Chao1 | Depth: 10–20 – 0–10 cm | -191.93 | -683.13 to 299.26 | 0.4224 |

| TS 4. Effects of environmental factors on Methanocellaceae abundance (ANOVA). | | | | | |
| --- | --- | --- | --- | --- | --- |
| Factor | df | Sum Sq | Mean Sq | F value | p-value |
| Wetland.Type | 1 | 3138 | 3137.8 | 16.63 | 0.0006 |
| Season | 1 | 74 | 74.0 | 0.39 | 0.54 |
| Depth | 1 | 270 | 270.3 | 1.43 | 0.25 |
| Residuals | 20 | 3775 | 188.7 | — |  |

| TS 5. Seasonal variation of environmental parameters within each wetland type (T-test) | | | | | | | |
| --- | --- | --- | --- | --- | --- | --- | --- |
| Variable | Grouping factor | Subset | Mean (Group 1) | Mean (Group 2) | t | df | p-value |
| pH | Season | Permanent | Dry: 5.32 | Rainy: 5.63 | -1.08 | 3.94 | ns |
| Eh | Season | Permanent | Dry: 248.8 | Rainy: 231.0 | 0.54 | 2.24 | ns |
| Soil moisture | Season | Permanent | Dry: 43.0 | Rainy: 44.1 | -0.21 | 3.27 | ns |
| pH | Season | Seasonal | Dry: 5.09 | Rainy: 5.13 | -0.09 | 4.00 | ns |
| Eh | Season | Seasonal | Dry: 351.2 | Rainy: 290.7 | 3.52 | 3.95 | 0.0249 |
| Soil moisture | Season | Seasonal | Dry: 31.0 | Rainy: 35.1 | -1.82 | 3.81 | ns |
| Eh | Wetland type | All samples | Permanent: 239.9 | Seasonal: 320.9 | -3.70 | 9.99 | 0.0041 |
| Soil moisture | Wetland type | All samples | Permanent: 43.5 | Seasonal: 33.0 | 3.99 | 8.21 | 0.0038 |
